# Raphe serotonergic neurons modulate genioglossus corticomotor activity in intermittent hypoxic rats

**DOI:** 10.1186/1465-9921-15-76

**Published:** 2014-07-08

**Authors:** Jiao Su, Wei Wang, Longfeng Sun, Ting Li, Delei Kong, Jian Kang

**Affiliations:** 1 Institute of Respiratory Disease, The first Hospital of China Medical University, 155 Nanjing Northern St, 110001 Heping District, Shen Yang, China

**Keywords:** Intermittent hypoxia, Genioglossus, Raphe nuclei, Serotonin, Transcranial magnetic stimulation

## Abstract

**Background:**

Genioglossus activity is greater during wakefulness but decreases to a weaker state during sleep in obstructive sleep apnea syndrome (OSAS) patients, compared to healthy subjects. Previous studies suggested that the corticomotor control of the genioglossus was modified in OSAS patients. Intermittent hypoxia (IH), the typical pathophysiological change in OSAS, can induce genioglossus facilitation. The serotonergic neurons of the raphe dorsal (DRN) and magnus nuclei (RMg) are responsive to hypoxia and play important roles in the control of the genioglossus. However, it remains unknown whether DRN and RMg serotonergic neurons are responsible for the facilitated corticomotor activity of the genioglossus during IH. This study explored the influence of IH on the corticomotor activity of the genioglossus by transcranial magnetic stimulation (TMS), and the role of DRN and RMg serotonergic neurons in this effect.

**Methods:**

Rats were exposed to IH and divided into two groups. In one group, anti-SERT-SAP was microinjected into the DRN and RMg respectively to kill serotonergic neurons. In the other group, artificial cerebrospinal fluid (ACSF) was injected. Comparisons were conducted between the two groups during four weeks of IH and four weeks after IH.

**Results:**

Compared to the corresponding ACSF-injected group, the DRN lesion group and RMg lesion group showed longer TMS latencies and lower amplitudes during IH from the 1^st^ to the 28^th^ day. After 28 days of IH, longer latencies and lower amplitudes were seen only in the DRN lesion group.

**Conclusion:**

These results indicate that DRN and RMg serotonergic neurons play different roles in the facilitation of genioglossus corticomotor activity induced by IH.

## Background

Obstructive sleep apnea syndrome (OSAS) is characterized by recurrent upper airway obstruction and intermittent hypoxia (IH) during sleep, but it does not occur while awake. The genioglossus is an extrinsic tongue muscle and its contraction results in opening the upper airway in the anterior-posterior dimension. Compared with healthy subjects, OSAS patients have greater genioglossus activity during wakefulness, but weaker activity while sleeping [1]. The waking-related increased genioglossus activity, which maintains upper airway patency, is hypothesized as a neuromuscular compensation for an anatomically small and more collapsible upper airway in OSAS patients. Series and Wang et al. confirmed the neuromuscular compensation of genioglossus in OSAS patients during wakefulness [2,3]. Identifying the mechanisms involved in the increased genioglossus activity of awake OSAS patients is important to understand the neuromuscular compensation of the genioglossus and to guide neuro-pharmacological interventions aimed at preventing the collapse of the upper airway. It has been reported that IH, the typical pathophysiological change in OSAS, can induce the facilitation of genioglossus activity [4]. A previous study in awake rats suggested that genioglossus motor-evoked potentials (MEPs) of transcranial magnetic stimulation (TMS) increased during IH [5]. However, it remains unknown how IH influences the central control of the genioglossus.

Alterations in the serotonin (5-HT) system have been suggested to be related to upper airway obstruction in animals. Reid and Rand were the first to note that intravenous administration of 5-HT caused apnea in cats [6]. Subsequently, Hilaire et al. demonstrated that intraperitoneal injection of L-tryptophan induced the activation of 5-HT biosynthesis in cerebrospinal fluid and abolished genioglossus activity in anaesthetized newborn rats [7]. Zhong et al. concluded that intracerebroventricular injection of 5-HT or 5-HT _2A/2C_ agonists can increase genioglossus activity in rats [8]. Sood and his colleagues further observed that genioglossus activity was increased by 5-HT at the hypoglossal motor nucleus in anesthetized rats [9]. Raphe 5-HT neurons, as respiratory premotor neurons, are subdivided into two main groups. One group is located in the pons/mesencephalon, and includes the dorsal raphe nucleus (DRN) and the median raphe nucleus. The other group consists of the caudal raphe nuclei, which mainly includes the raphe magnus (RMg), the raphe obscurus, raphe pallidus nuclei and the parapyramidal region. Many regions of the brainstem are known to project to hypoglossal motoneurons, including the DRN, the nucleus subcoeruleus, laterodorsal and peduculopontine tegmental nuclei, the pontine and medullary reticular formation, etc [10]. Barker et al. have recently found that the majority of 5-HT neurons projecting to hypoglossus motoneurons are located in the RMg (26%) and the parapyramidal region (45%) [11]. Thus, the 5-HT neurons of the DRN and RMg may take part in the central control of genioglossus activity. McKay and his colleagues have observed that IH can elicit the long-term facilitation of the respiratory-modulated activity of the genioglossus in a neonatal rat, but the long term-facilitation of genioglossus can’t be observed in the sustained hypoxia condition [12]. Sustained hypoxia can result in decreased 5-HT cell expression in the DRN of rabbits [13] and Fos expression in the 5-HT neurons of the RMg and DRN in rats [14]. Ohliger-Frerking demonstrated that DRN 5-HT neurons are hyper-excitable in obese Zucker rats, which are often used as animal models of OSAS [15]. McNamara et al. have observed that IH could result in a higher 5-HT concentration in DRN of adult rabbits [16]. Nevertheless, it remains unknown whether the increased genioglossus responses during IH is modulated by 5-HT neurons in the DRN and/or RMg.

Single-pulse TMS can be used to explore the corticospinal pathway of the genioglossus [17]. For a given muscle, the corticospinal activation process can be characterized by the effects of stimulating conditions on the characteristics (amplitude and latency) of MEP. Wang et al. observed increased genioglossus neuromuscular activity in awake OSAS patients by TMS [2,3]. Therefore, the present study aimed to use this technique to explore the role of 5-HT neurons of the DRN and RMg in the central control of the genioglossus during and after IH in awake rats.

## Methods

### Animals

Specific Pathogen Free adult male Wistar rats were provided by the laboratory animal center of China Medical University, ranging from 250 g to 300 g. The rats had free access to water and food and were housed under controlled conditions (temperature 24 ± 2°C, relative air humidity 40%) with a 12:12 h light-dark cycle (lights on at 8:00 am and lights off at 8:00 pm). All procedures were performed in accordance with the National Institute of Health Guide for Care and Use of Laboratory Animals and were approved by the animal Ethics and Use Committee of China Medical University.

### Intermittent hypoxia

The rats were randomly divided into two groups: IH and normoxic (NO) groups. The rats in IH group were subjected to oxycycler (Oxycycler model A84XOV; BioSpherix, NY) hypoxia (10% O2 in N2 for 45 s) and normoxia (21% O2 in N2 for 60 s) every 188 s for 8 h/d (from 8 am to 4 pm), for 4 consecutive weeks. The rats in NO group were subjected to alternating cycles of air under identical experimental conditions in parallel. The capacity of the polypropylene animal chamber (A-15274-P-EVAC, BioSpherix) was 15”W × 20”D × 20”H. The O_2_ concentration was continuously measured by an O2 analyzer and was changed by a computerized system controlling gas outlets.

### Drug

Anti-SERT-SAP (1 μM in artificial cerebrospinal fluid, ACSF; Advanced Targeting Systems, San Diego, CA, USA) is a chemical conjugation of a monoclonal antibody to the fourth extracellular domain of the serotonin re-uptake transporter and the ribosome-inactivating protein - saporin. Nattie et al. have demonstrated that the exposure of medullary raphe neuron cultures to anti-SERT-SAP lead to the killing of 5-HT neurons without an effect on the other neurons on the same plates in vitro [18]. ACSF (pH7.35-7.45) was equilibrated with 95% O2 and 5% CO2, and contained NaCl 125 mM, KCl 3 mM, KH2PO4 1 mM, CaCl2 2 mM, MgSO4 1 mM, NaHCO3 25 mM and D-glucose 30 mM [19].

### Surgery

The rats were subjected to general anesthesia, and any additional anesthesia during surgery was given by inhalation (isoflurane 0.2-2%). Effective anesthesia was judged by abolition of the pedal withdrawal and corneal blink reflexes. The rats were placed in a stereotaxic apparatus (68003, RWD Life Science, China). The head was shaved, and the skin was sterilized with alcohol and incised. Each rat in the experimental group received two injections of anti-SERT-SAP (0.1 μl, 1 μM) in the regions of interest. The rats in the control group underwent the same procedures except that ACSF was microinjected instead of anti-SERT-SAP. The details of the above methods have been described previously [20]. The coordinates for the placement of the DRN were 7.20 mm-8.04 mm posterior to bregma in the midline, and 6.4 mm below the dorsal surface of the skull. The coordinates of the RMg were 10.52 mm-11.30 mm from bregma in the midline, and 10.4 mm below the dorsal surface of the skull [21]. The injections were made by using a 0.5-μl Hamilton microsyringe with a 28-gauge dental needle and were performed with a microinjector machine (model 310, Stoelting, IL). Each injection last 4 min; the microsyringe was left in situ for another 5 min after microinjection to limit efflux of injection from the microsyringe. Then the incision was sutured.

### TMS-MEP

TMS was performed on awake and non-sedated rats. The rats were positioned on a wooden board and their heads, bodies and limbs were restrained. Then single-pulse TMS was performed by a Magstim 200 stimulator (Magstim, Whiteland, Dyfed, UK) with a 70 mm figure-eight coil. The coil was held against the rat’s head. The TMS response of the genioglossus corticomotor area was described in terms of the corresponding MEP amplitude and latency. The optimal coil position was defined as that with the best response to TMS (the highest MEP amplitude and the shortest MEP latency). Briefly, the optimal stimulation site was 3.0-5.0 mm rostral to bregma, and 2.0-4.0 mm lateral from the midline. The site was clearly marked with indelible ink, and the position of the coil was kept constant at the stimulation site by using a high-precision multipositional support consisting of two articulated arms. For each position, five stimuli were applied at least 30 s intervals and averaged for mean MEP response. All TMS were delivered at the end of normal expiration in awake rats at 6:00 pm. The respiratory phase was determined by the detection of abdominal movement.

Genioglossus MEPs were recorded by inserting a concentric needle electrode (NM-131 T, NIHON Kohden, Japan) into the genioglossus muscle belly repeatedly on each recording day. MEP signals, with the motor threshold of 100%, were amplified (with filters at 10Hz to 5 kHz), digitized and recorded using a computer software package (AxoScope software 9.0, Axon Instruments, Inc., USA). MEP latency was defined as the time up to the first deflection from baseline following TMS, and MEP amplitude was measured from peak to peak TMS response.

### Experimental protocol

**Figure 1:**
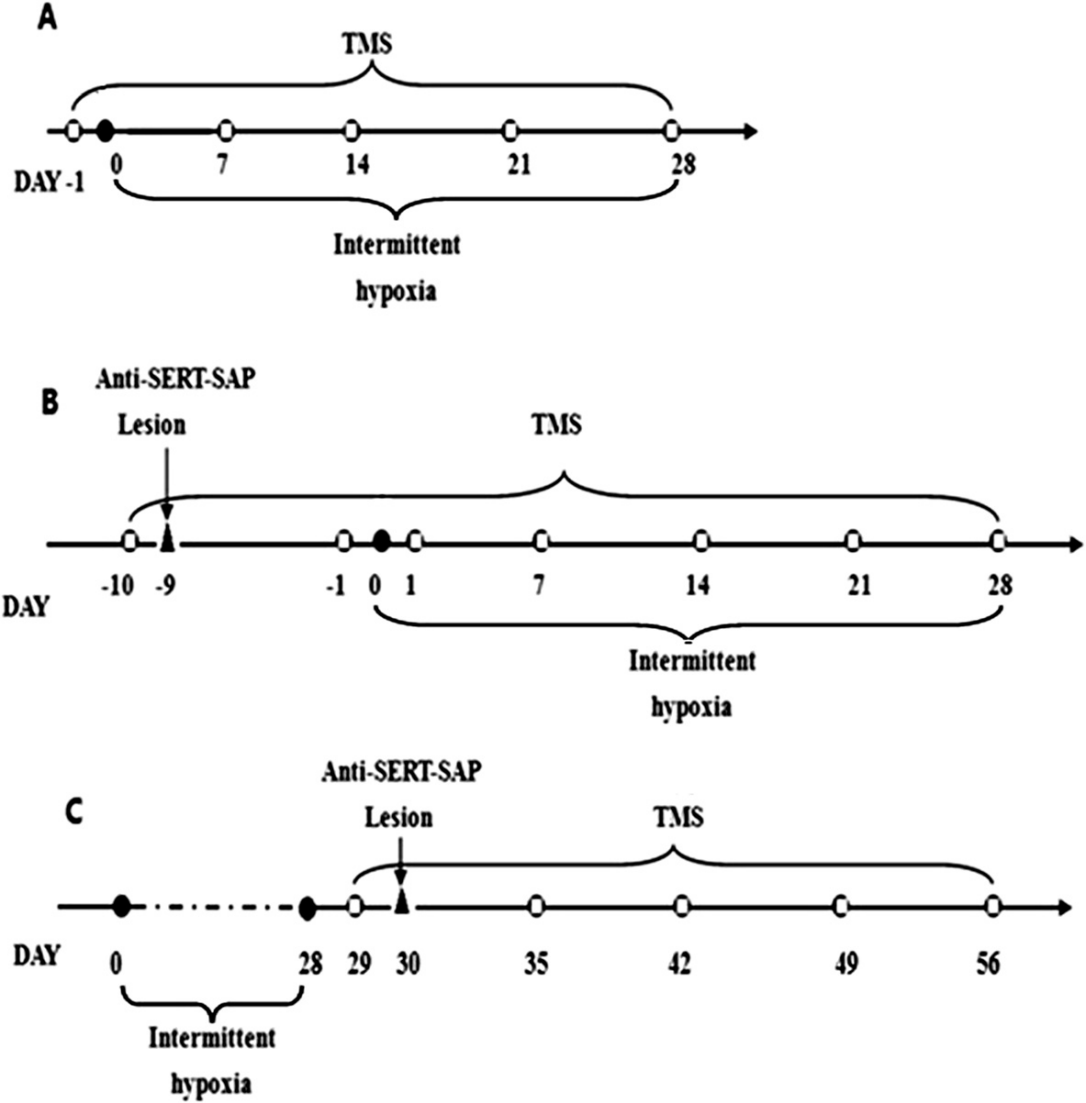
**Schematic diagram of the experimental protocol. **: TMS. **Panel A** represents the procedure of the first series of experiments, **panel B** represents the procedure of the second series of experiments, **panel C** indicates the procedure of the third series of experiments.

The experiments were performed on 92 rats.In the first series of experiments, the rats were divided into two groups: a IH group (n = 10) and a NO group (n = 10). The TMS schedule is shown in Figure 1A.In the second series of experiments, the rats were randomly divided into four groups and then exposed to IH: DRN lesion during IH (n = 10) and DRN control during IH (n = 10); RMg lesion during IH (n = 10) and RMg control during IH (n = 8). IH was performed 9 days after lesions. The TMS schedule is shown in Figure 1B.In the third series of experiments, the rats were exposed to IH for 4 weeks and then randomly divided into four groups: DRN lesion after IH (n = 10) and DRN control after IH (n = 6); RMg lesion after IH (n = 10) and RMg control after IH (n = 8). The TMS schedule is shown in Figure 1C.

### 5-HT immunohistochemistry

The number of rats used for 5-HT immunohistochemistry was respectively 20 in DRN 5-HT neurons lesion group, 16 in DRN control group, 20 in RMg 5-HT neurons lesion group, and 16 in RMg control group. The effectiveness of the specific lesions of the DRN and RMg 5-HT neurons with anti-SERT-SAP were verified by immunohistochemistry. The rats were deeply anesthetized with 10% chloral hydrate and perfused using PBS (PH 7.4) and 4% paraformaldehyde (4% in 0.1 mol/L PBS, pH 7.4). The brain was removed and postfixed in 4% paraformaldehyde overnight at 4°C, dehydrated in ethanol, cleared in dimethylbenzene and embedded in paraffin. Then the brain was cut coronally to 5 μm serial sections in a microtome (RM2015, Leica). The tissue sections were placed on polylysine-coated glass slides, deparaffinized in dimethylbenzene, rehydrated in ethanol, rinsed in distilled water for 5 min, and treated with microwave for antigen retrieval in citrate buffer (10 mM, PH 6.0) for 5 min three times. Endogenous peroxidase was quenched with 3% H_2_O_2_ for 10 min and nonspecific binding was blocked by normal goat serum for 60 min. The sections were incubated with polyclone rabbit anti-5-HT antibody (1:800, Abcam) overnight at 4°C. After being washed in PBS, the sections were incubated with the secondary antibody for 60 min (HRP/DAB Detection IHC Kit, Abcam) and placed in streptavidineperoxidase complex for 50 min. The labeled neurons were visualized by a 10-min incubation with 3,3′-diaminobenzidine at room temperature. The sections were counterstained with haematoxylin to enhance the visualization of the tissue architecture and cytological details. The sections processed without the addition of primary antibody served as negative controls; 5-HT-immunoreactive (5-HT-ir) staining was not found in the negative control sections. Cells detected with brown surface membranes and/or cytoplasm were considered immunoreactive for 5-HT.

Six serial sections in areas of interest from each rat were examined. For each section, the number of 5-HT-ir cells were checked bilaterally and quantified by a computerized system, which included a microscope (Olympus BX51, Japan) equipped with a CCD micrographic system (U-CMAD3, Japan). To evaluate whether our microinjections produced distant effects, we also counted 5-HT-ir cells of the RMg in the DRN specific lesion groups, and quantified 5-HT-ir cells of the DRN and the raphe obscurus nucleus in the RMg specific lesion groups. For each rat, all sections were averaged and the mean value was considered to be the estimated number of 5-HT-ir cells for the section.

### Statistical analysis

The results are reported as means ± SD. The numbers of somatic cell profiles of 5-HT-ir neurons were compared using an unpaired *t*-test between the ACSF-injected group and the anti-SERT-SAP-injected group. For the analysis of genioglossus MEP latencies and amplitudes among different groups, we performed a repeated measures multivariate analysis of variance (MANOVA), with three factors: treatment (lesions), time (day) and stimulus (IH). One-way ANOVA was used to analyze significance interactions. The Duncan test was performed for multiple comparisons. The statistical analysis was performed using SPSS 16.0 for Windows. *P* <0.05 was considered statistically significant.

## Result

### 5-HT immunohistochemistry

**Figure 2:**
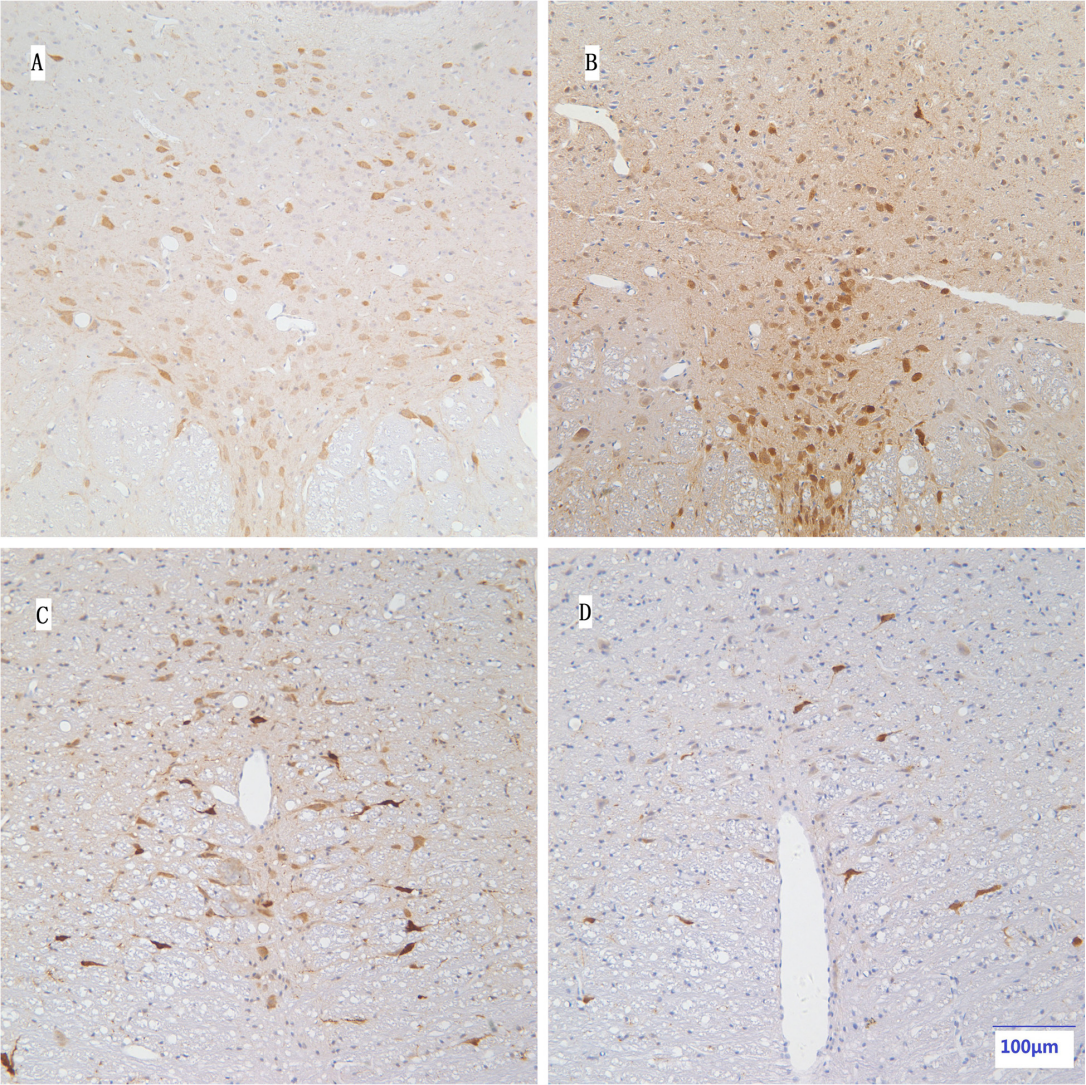
**Specific lesion of 5-HT neurons in the DRN and RMg.** A representation of 5-HT-ir in the DRN **(A, B)** and RMg **(C, D)** between the ACSF-injected group **(left column; A, C)** and the anti-SERT-SAP-injected group **(right column; B, D)**. Magnifications: panels **A**, **B**, **C**, **D** (bar scale 100 μm).

**Figure 3:**
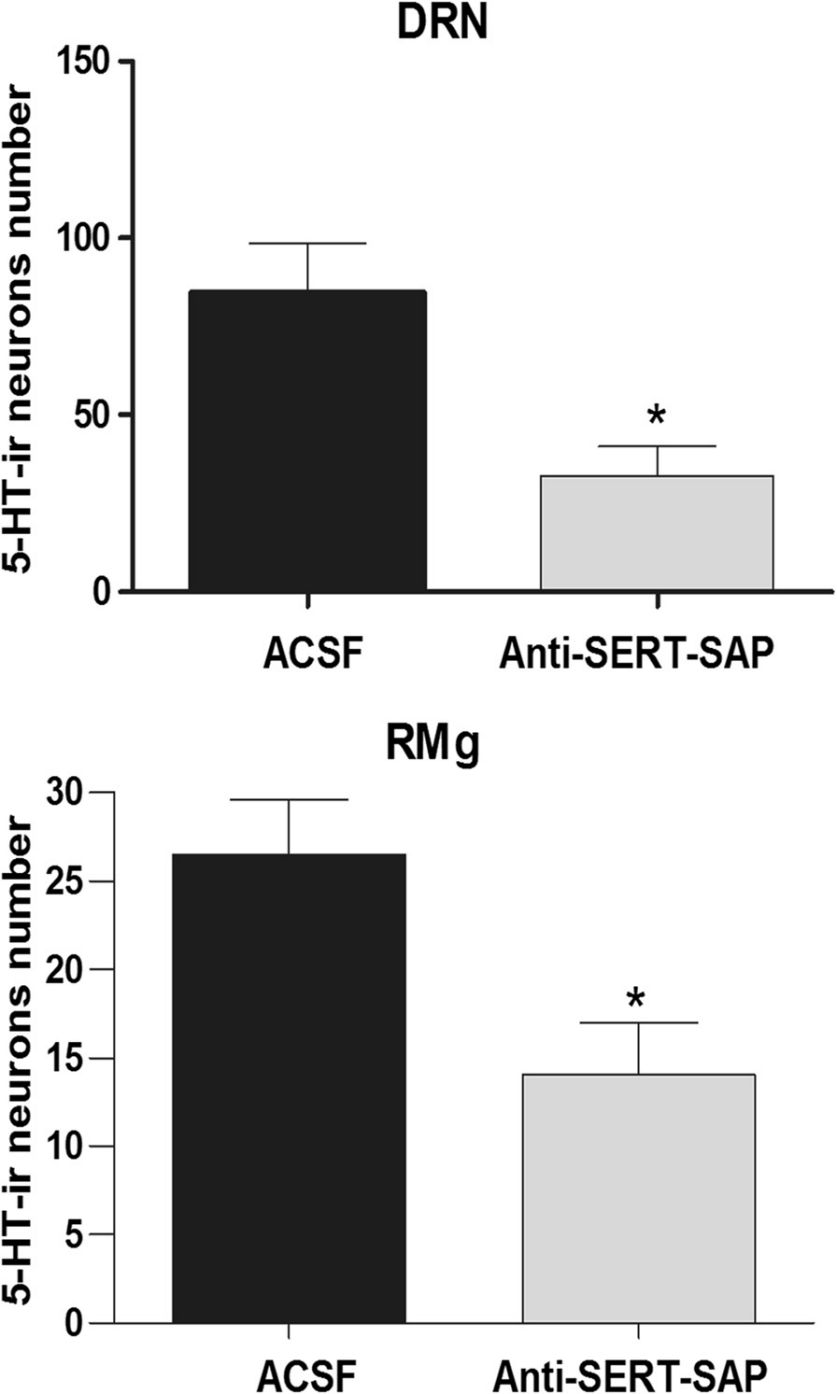
**Comparisons of DRN (above) and RMg (below) 5-HT-ir neurons between the ACSF-injected group (black column) and the anti-SERT-SAP-injected group (gray column).** *P < 0.05 indicates a significant difference.

When compared with the ACSF-injected groups, the group which received anti-SERT-SAP exhibited specific lesion of DRN and RMg 5-HT neurons, and the number of 5-HT-ir cells was significantly reduced in anti-SERT-SAP-injected group (DRN: 80.60 ± 6.18/section for the ACSF-injected group and 32.67 ± 4.19/section for the anti-SERT-SAP-injected group, *P* < 0.05; RMg: 26.49 ± 3.09/section for the ACSF-injected group and 14.03 ± 2.93/section for the anti-SERT-SAP-injected group, *P* < 0.05) (Figures 2 and 3). The other regions of the brainstem were not affected.

### The MEP latencies and amplitudes of genioglossus in different groups

**Figure 4:**
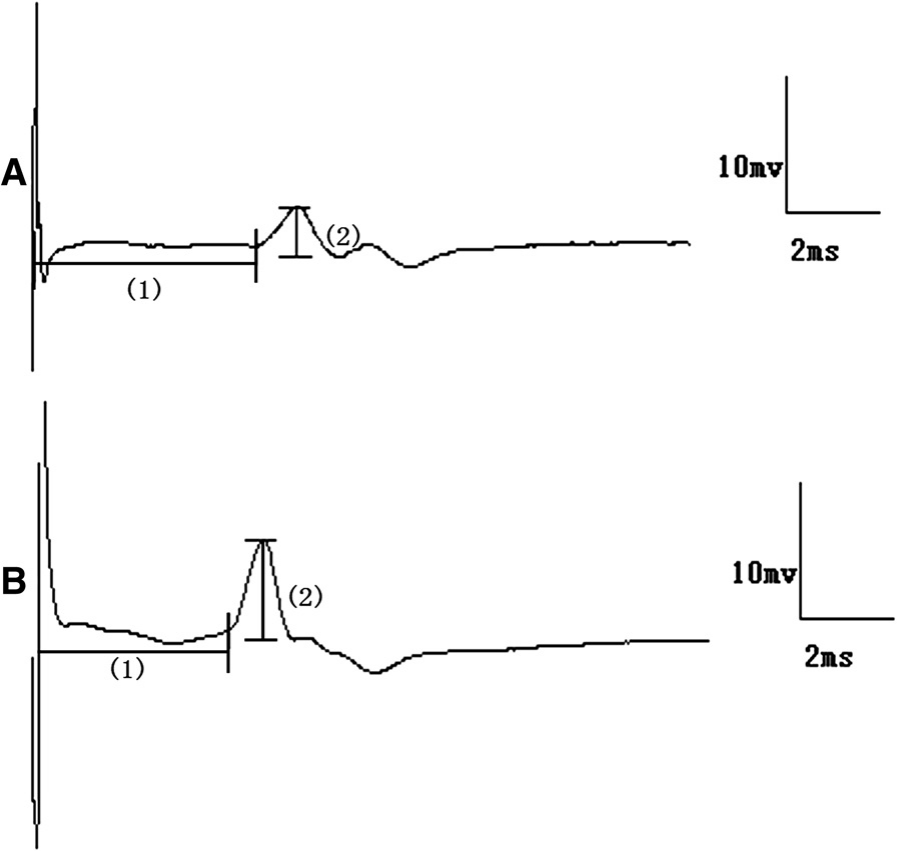
**Typical genioglossus motor evoked potential during NO (a) and IH (b).** Principal features are latency (1), amplitude (2).

**Figure 5:**
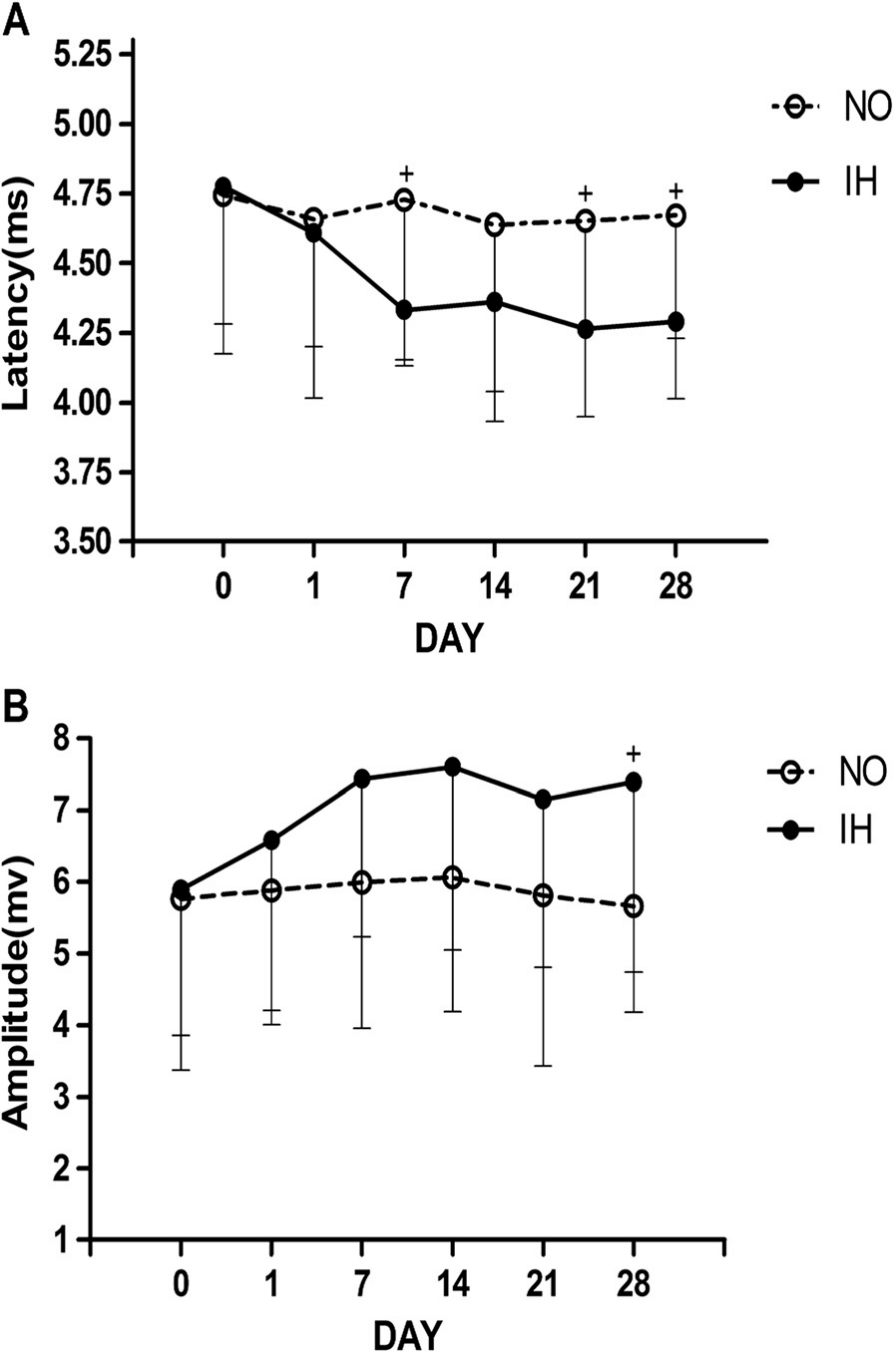
**The MEP latency (A) and amplitude (B) of genioglossus in the NO and IH groups.** + Indicates a significant difference between the NO and IH groups at the same measurement time (P < 0.05).

**Figure 6:**
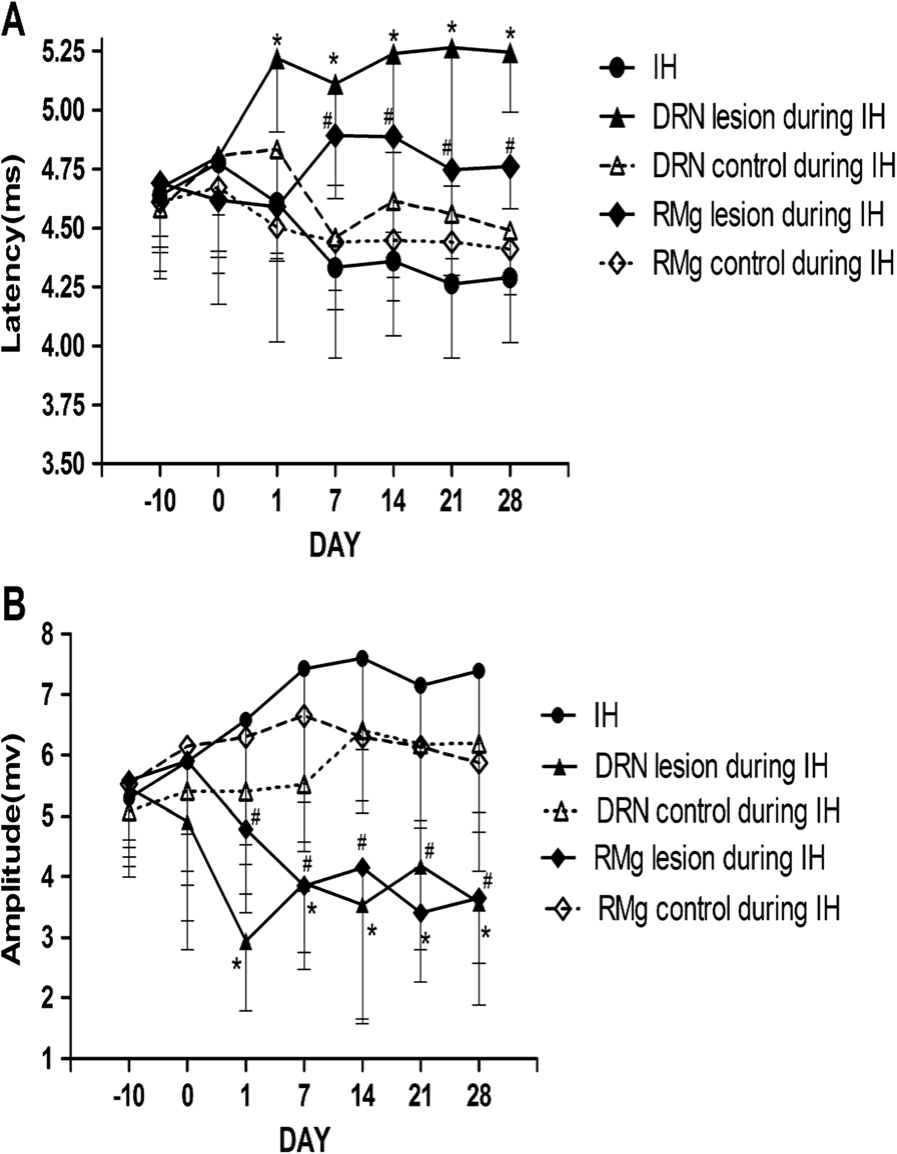
**The MEP latency (A) and amplitude (B) of genioglossus in groups in the second experimental series.** *Indicates P < 0.05 between the DRN lesion during IH group and the DRN control during IH group at the same measurement time; # Indicates P < 0.05 between RMg lesion during IH group and RMg control during IH group at the same measurement time.

**Figure 7:**
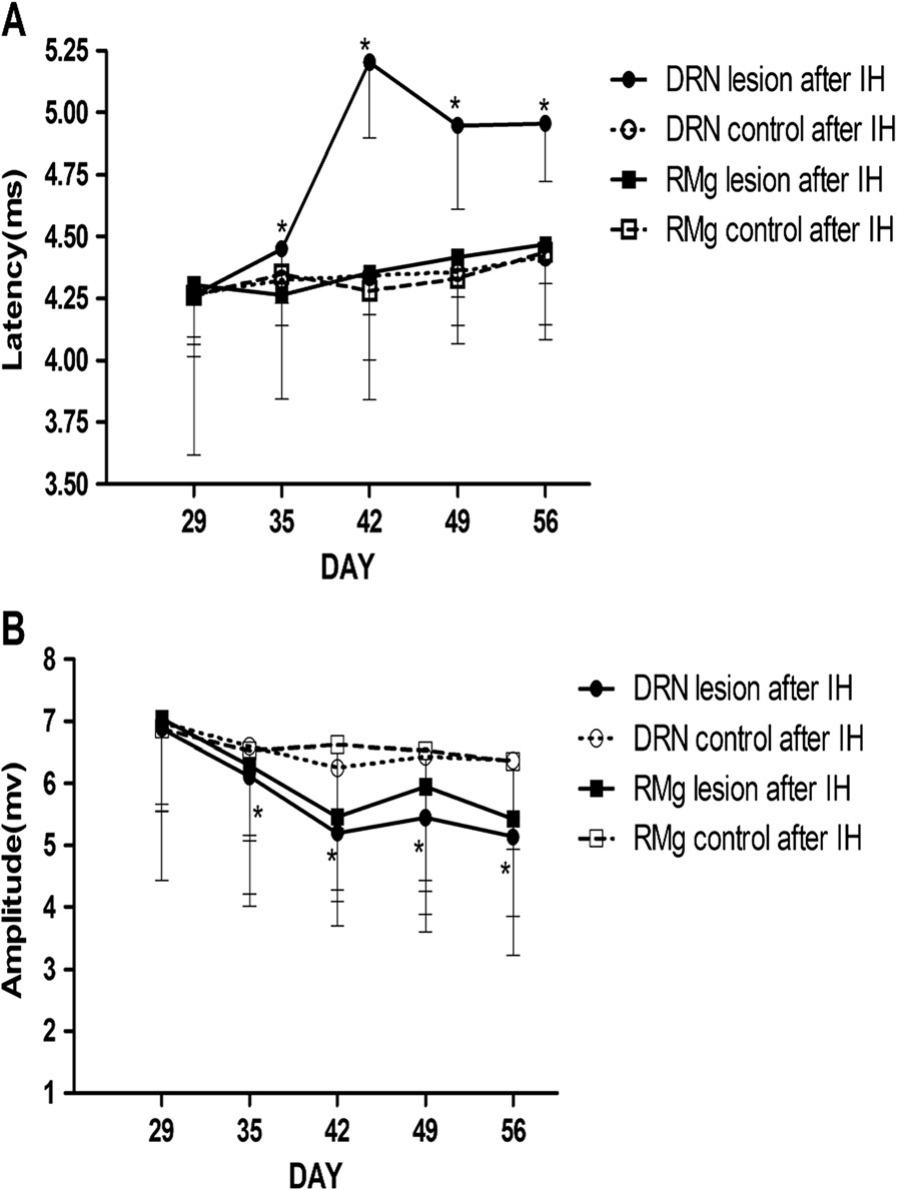
**The MEP latency (A) and amplitude (B) of genioglossus in groups of the third experimental series.** *Indicates P < 0.05 between the DRN lesion after IH group and the DRN control after IH group at the same measurement time.

Figure 4 depicts typical examples of genioglossus responses to TMS in the NO and IH groups. In the first series of experiments, the IH group exhibited decreased genioglossus MEP latencies and increased amplitudes after 7-day of IH, compared with the NO group. Significant differences in latency were observed on the 7^th^, 21^st^ and 28^th^ day of IH, and significant differences in amplitude were observed on the 28^th^ day of IH (Figure 5). In the second series of experiments, compared with the corresponding control group, longer MEP latencies and lower MEP amplitudes were observed in the DRN lesion during IH group and RMg lesion during IH group respectively from 1^st^ day to 28^th^ day of IH (Figure 6). In the third series of experiments, compared with the DRN control after IH group, the DRN lesion after IH group showed longer latencies and lower amplitudes from 7^th^ day to 28^th^ day after IH. However, no significant MEP difference was observed between the RMg lesion after IH group and the RMg control after IH group (Figure 7).

## Discussion

This study yielded two major findings. First, IH can elicit an increase in genioglossus corticomotor response to TMS that is characterized by shorter MEP latencies and higher amplitudes. Such a genioglossus corticomotor facilitation phenomenon is in accord with the data on OSAS patients published by Wang et al. [3]. Second, lesioning DRN 5-HT neurons significantly attenuated the increased genioglossus TMS responses during and after IH, while lesioning RMg 5-HT neurons only had a similar effect during IH. McKay and his colleagues have observed that IH can elicit the long term-facilitation of the respiratory-modulated activity of the genioglossus in a neonatal rat [12]. Mahamed et al further reported that apnea-induced long-term facilitation of hypoglossal motoneurons was 5-HT-dependent [22]. McNamara et al. have demonstrated that the IH could result in a higher 5-HT concentration in DRN of adult rabbits [16]. This study is the first to explore the different roles of DRN and RMg 5-HT neurons in modulating genioglossus activity in IH rats at the level of the corticomotor cortex using TMS technology.

The genioglossus shows both phasic and tonic activities. In the present study, we measured genioglossus MEP at the end of normal expiration to eliminate the influence of different respiratory phases. We found that IH elicited a facilitation of genioglossus TMS responses. This phenomenon also has been found in OSAS patients during wakefulness [3]. This raises the question of where is the site involved in IH-induced alteration of genioglossus activity. A preactivation of motoneurons is the most commonly accepted mechanism to explain the facilitated response to TMS for a given muscle. The genioglossus is a special muscle because it is not only involved in respiration, but also has nonrespiratory function, such as swallowing. The total motor outflow to the genioglossus is the sum of the respiratory and nonrespiratory inputs to hypoglossal motoneurons. Therefore, the genioglossus MEP reflects the integrate activities of respiratory premotor neurons and the corticospinal tract. If the increased genioglossus TMS responses during IH are mainly induced by the corticospinal tract, the MEP of other skeletal muscles should be also affected during IH. In fact, Civardi et al found no difference in first dorsal interosseous muscle MEP latency and amplitude between OSAS patients and normal subjects when awake, but the central silent period was longer in patients than in controls [23]. These results suggested a widespread depression of the corticomotor area, except for the pharyngeal corticomotor area in OSAS patients. In fact, respiratory premotor neurons activity is expected to be influenced by hypoxia. Our results suggest that the increased genioglossus TMS responses during IH mainly reflect the influence of respiratory input. These changes are possibly related to the adaptive mechanism of the respiratory system. Ling et al. has confirmed that IH could elicit 5-HT-dependent facilitation of diaphragm in rats [24] and his colleagues further found that the acute IH-induced genioglossus facilitation was dependent on 5-HT_2_ and N-methy-D-aspartate receptors [25].

DRN and RMg areas of the raphe nuclei modulate the genioglossus activities by 5-HT neurons. We found that the facilitation of genioglossus activity during IH can be abolished by killing DRN and RMg 5-HT neurons. However, only the lesioning of DRN 5-HT neurons can reverse IH-induced facilitation of genioglossus TMS responses after IH. The different effects of DRN and RMg 5-HT neurons on genioglossus TMS responses might result from their different anatomic and neural connections. RMg serotonergic neurons directly project to hypoglossus motoneurons [11,26]. As a central chemoreceptor, it participates in the facilitation of genioglossus induced by IH. Also, Gargaglioni et al have found that RMg serotonergic neurons do not participate in the ventilation during air breathing, but contribute to the ventilatory response to hypoxia [27]. It is correspondence with our findings. On the other hand, no matter IH exists or not, DRN serotonergic neurons always induce the increased activity of genioglossus. This may be related to the fact that DRN participates in both the cortical arousal system and the hypoglossal motoneurons [10,28-30]. The increased genioglossus activity has been observed across sleep-wake cycle when the 5-HT_1A_ receptor of DRN descending neurons was suppressed [31]. Our results further confirm that DRN and RMg 5-HT neurons are both involved in genioglossus facilitation during IH. Once the facilitation of central neural circuit of genioglossus has been elicited by IH, 5-HT neurons of the RMg withdrew from this adaptative mechanism while 5-HT neurons of the DRN continued to play an important role.

As for the method, anti-SERT-SAP was microinjected 9 days prior to the administration of IH in the second series of experiments. Previous studies have specifically used anti-SERT-SAP to kill the 5-HT-containg neurons. Nattie et al and Dias et al have concluded that the killing of 5-HT neurons was significantly delayed for 4 days after the administration of anti-SERT-SAP, and the maximum lesion was on the days 7 to 10 [18,20]. Nattie et al. have also observed that the exposure of medullary raphe neuron cultures to anti-SERT-SAP leads to the killing of serotonergic neurons without any effects on the other neurons in vitro [18]. In our study, the method was a repeat from the previous statement [18,32,33]. Furthermore, immunohistochemistry was applied to verify the specific lesions of the 5-HT neurons. Using a similar approach, Da Silva GS et al. reported that the 5-HT neurons were reduced by 37%-38% and focal killing (12.3 to 12.8 mm posterior to the bregma) was about 44%-68% after specific lesions by anti-SERT-SAP [32,33]. Dias et al. reported that the reduction of 5-HT neurons was approximately 35%, and 5-HT neurons in the raphe magnus had a 50% reduction via injection of the anti-SER-SAP [20]. In the present study, we detected the reduction in 5-HT neurons (about 50% cell less) at the level of 7.20 mm-8.04 mm in the DRN and 10.52 mm-11.30 mm in the RMg, and the reduction was near the microinjection sites. Thus, these data showed a large reduction in the amount of intact 5-HT neurons.

The limitations of the present study are as follows. First, there is potential criticism for performing TMS with a 70 mm figure-eight coil, because its effect may spread to other areas besides the genioglossus corticomotor area. In order to avoid this effect, the genioglossus MEP was recorded with a concentric needle electrode in our study. Therefore, the TMS responses reflected real genioglossus activity. Second, this study was performed only with male rats and did not explore the influence of gender. The occurrence of OSAS exhibits gender differences, and it is assumed that sex hormone may play a role in the etiology of OSAS. Thus, we excluded the influence of gender on the control of genioglossus activity. Third, it is interesting to note that the decreased TMS response lasted for many days after cessation of IH exposure and slowly returned to the baseline level in the present study. This may be associated with the persistence of changes after a period of chronically administered IH. Determining the specific mechanisms will require further study. Fourth, hypercapnia is another important pathophysiological change in obstructive sleep apnea patients and it also has an important effect on 5-HT neurons. The effect of CO_2_ and IH on 5-HT neurons deserves further study. In order to observe the influence of IH, we especially excluded the influence of CO_2_ on the control of the genioglossus activity in this study.

## Conclusions

In summary, these results indicate that DRN and RMg 5-HT neurons participate in IH-induced facilitation of genioglossus TMS responses. We believe that the augmented genioglossus activity during IH represents a neuromuscular compensation aimed at maintaining upper airway patency. The different role of DRN and RMg 5-HT neurons during different periods of IH should be taken into account when the compensation mechanism is explored, and this may be helpful in finding a new method for OSAS treatment. The increased TMS responses of the genioglossus corticomotor area undoubtedly require the entire respiratory neural network. Different 5-HT receptor subtypes have different roles in modulating genioglossus in OSAS patients. The influence of the sleep-wake cycle also should be adequately considered. Further investigations will be required to fully understand the role of different parts of 5-HT neurons.

## Abbreviations

OSAS: Obstructive sleep apnea syndrome; IH: Intermittent hypoxia; GG: Genioglossus; MEP: Motor-evoked potential; 5-HT: 5-hydroxytryptamine/serotonin; DRN: Dorsal raphe nucleus; RMg: Raphe magnus nucleus; TMS: Transcranial magnetic stimulation; NO: Normoxia; ACSF: Artificial cerebrospinal fluid; EMG: Electromyography.

## Competing interests

The authors declare that they have no competing interests.

## Authors’ contributions

JS carried out the animal experiments, analyzed data, interpreted the data of experiments, drafted manuscript. WW conceived of the study, participated in the design of the study, edited and revised manuscript. LS carried out the 5-HT immunohistochemistry, drafted manuscript. TL performed the statistical analysis, drafted manuscript. DK analyzed data, drafted manuscript. JK participated in the design of the study, interpreted the results of experiments, revised of manuscript. All authors read and approved the final manuscript.
